# Shedding Light on the Enigmatic TcO_2_ ⋅ *x*H_2_O Structure with Density Functional Theory and EXAFS Spectroscopy**

**DOI:** 10.1002/chem.202202235

**Published:** 2022-09-23

**Authors:** Augusto F. Oliveira, Agnieszka Kuc, Thomas Heine, Ulrich Abram, Andreas C. Scheinost

**Affiliations:** ^1^ Institute of Resource Ecology Helmholtz-Zentrum Dresden-Rossendorf (HZDR) Forschungsstelle Leipzig Permoserstr. 15 04318 Leipzig Germany; ^2^ Theoretische Chemie Technische Universität Dresden Bergstr. 66c 01062 Dresden Germany; ^3^ Department of Chemistry Yonsei University 50 Yonsei-ro, Seodaemun-gu Seoul 03722 (Republic of Korea; ^4^ Institut für Chemie und Biochemie Anorganische Chemie Freie Universität Berlin Fabeckstr. 34–36 14195 Berlin Germany; ^5^ Institute of Resource Ecology Helmholtz-Zentrum Dresden-Rossendorf (HZDR) Bautzner Landstr. 400 01328 Dresden Germany; ^6^ The Rossendorf Beamline (ROBL) European Synchrotron Radiation Facility (ESRF) 71, Avenue des Martyrs Grenoble 38043 France

**Keywords:** chain structures, density functional calculations, EXAFS spectroscopy, nuclear waste management, technetium

## Abstract

The β‐emitting ^99^Tc isotope is a high‐yield fission product in ^235^U and ^239^Pu nuclear reactors, raising special concern in nuclear waste management due to its long half‐life and the high mobility of pertechnetate (TcO_4_^−^). Under the conditions of deep nuclear waste repositories, Tc is retained through biotic and abiotic reduction of TcO_4_^−^ to compounds like amorphous TcO_2_ ⋅ *x*H_2_O precipitates. It is generally accepted that these precipitates have linear (Tc(μ‐O)_2_(H_2_O)_2_)_*n*_ chains, with *trans* H_2_O. Although corresponding Tc−Tc and Tc−O distances have been obtained from extended X‐ray absorption fine structure (EXAFS) spectroscopy, this structure is largely based on analogy with other compounds. Here, we combine density‐functional theory with EXAFS measurements of fresh and aged samples to show that, instead, TcO_2_ ⋅ *x*H_2_O forms zigzag chains that undergo a slow aging process whereby they combine to form longer chains and, later, a tridimensional structure that might lead to a new TcO_2_ polymorph.

## Graphical abstract

**What shape are you in**? The structure of amorphous TcO_2_ ⋅ *x*H_2_O has been determined from DFT calculations and EXAFS measurements. Contrary to the generally accepted model, TcO_2_ ⋅ *x*H_2_O forms zigzag chains of *cis*‐Tc(μ‐O)_4_(H_2_O)_2_ units. A slow aging process results in increasing polymerization that might eventually lead to a new TcO_2_ polymorph. Formation of these phases with low solubility is highly relevant for the confinement of long‐lived ^99^Tc in deep nuclear waste repositories.

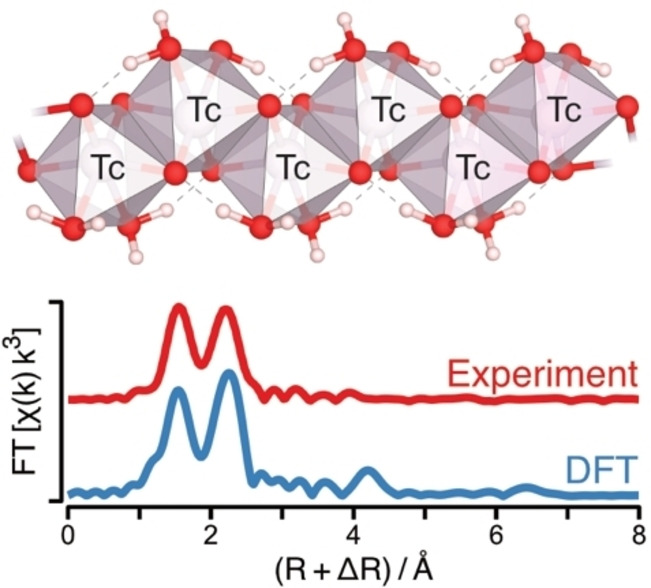

## Introduction

Technetium is the lightest element without a stable isotope and is almost completely artificial; only trace amounts are found in nature, formed from spontaneous fission in uranium minerals. ^99m^Tc (metastable) is of great importance in nuclear medicine as a γ‐ray emitter,^[1]^ while the β‐emitting ^99^Tc (ground state) has no practical use and raises special concern in nuclear waste management due to its long half‐life (ca. 2.1×10^5^ years) and relatively high formation yield (≥6 %) in ^235^U and ^239^Pu nuclear reactors.

In water and in absence of other complexing agents, Tc prevails in oxidation states VII and IV.^[2]^ Under nonreducing conditions, Tc^VII^ forms the soluble TcO_4_^−^ anion, which is highly mobile in the environment due to its weak interaction with adsorbent surfaces. Under the reducing conditions of natural, anoxic sediments as well as in nuclear waste repositories, however, numerous studies suggest that Tc precipitates as TcO_2_ ⋅ *x*H_2_O, either as colloids or associated with Fe^II^‐bearing mineral phases, which also act as catalysts for the Tc reduction.[^2^, ^3^] Understanding the Tc redox processes in these conditions is essential for evaluating the safety of nuclear disposal sites; however, to date, even the structure of the TcO_2_ ⋅ *x*H_2_O precipitates remains enigmatic and needs to be resolved.

Lukens et al.^[4]^ proposed that TcO_2_ ⋅ *x*H_2_O is formed by linear chains of equally spaced edge‐sharing TcO_6_ octahedra with terminal H_2_O ligands in the *trans* positions. In the same year, Vichot et al.^[5]^ arrived at similar conclusions, but argued that the structure would alternate shorter and longer Tc−Tc distances along the chain, based on data available for TcO_2_ crystals.^[6]^ However, these chain structures were based exclusively on EXAFS spectroscopy, which only provides radial interatomic distances and estimates of coordination numbers, and analogies to known crystal structures of other systems, like CoCl_2_ ⋅ 2 H_2_O and RuO_2_ ⋅ 2 H_2_O. In addition, in a recent work, Yalçintaş et al.^[3a]^ showed that both the chain model with equally spaced Tc atoms (similar to the model by Lukens et al.^[4]^) and the one with alternating Tc−Tc distances (similar to crystalline TcO_2_) could describe equally well their own EXAFS measurements of a fresh TcO_2_ ⋅ *x*H_2_O precipitate, thereby demonstrating that a conclusive characterization of TcO_2_ ⋅ *x*H_2_O based on EXAFS alone is impossible, even though EXAFS is, to the best of our knowledge, the only experimental technique applicable in this case due to the noncrystalline nature of TcO_2_ ⋅ *x*H_2_O.

In this work, in order to unambiguously assign the TcO_2_ ⋅ *x*H_2_O structure, we use density‐functional theory (DFT) to obtain energetically viable structural models. The results from the DFT modeling are then used in the interpretation of EXAFS spectra of fresh and aged samples, showing that TcO_2_ ⋅ *x*H_2_O precipitates are polymeric structures consisting of zigzag chains of TcO_6_ octahedra with H_2_O groups in a *cis* configuration. In addition, we show that these polymeric chains eventually combine laterally, forming a tridimensional structure that might evolve to a new, low‐energy TcO_2_ crystal phase.

## Results and Discussion

For the DFT modeling, our strategy was to derive infinite TcO_2_ ⋅ 2H_2_O chains from TcO_2_ crystal structures constructed from the crystallographic coordinates of the three known ReO_2_ polymorphs, namely α‐ReO_2_ (*P*2_1_/*c*),^[7]^ β‐ReO_2_ (*Pbcn*),^[8]^ and γ‐ReO_2_ (*P*4_2_/*mnm*).^[9]^ Although only one TcO_2_ phase has been characterized experimentally (almost identical to α‐ReO_2_),^[10]^ the use of ReO_2_ as initial reference is justifiable because Tc and Re have very similar crystal chemistry^[11]^ and, thus, TcO_2_ counterparts of all ReO_2_ polymorphs should be expected to form. The corresponding α‐, β‐, and γ‐TcO_2_ structures were obtained by replacing the Re atoms with Tc and performing full geometry optimizations, as described in the Experimental Section. Indeed, the optimized TcO_2_ structures turned out to be comparable to the ReO_2_ phases (Table S1 in the Supporting Information).

**Figure 1:**
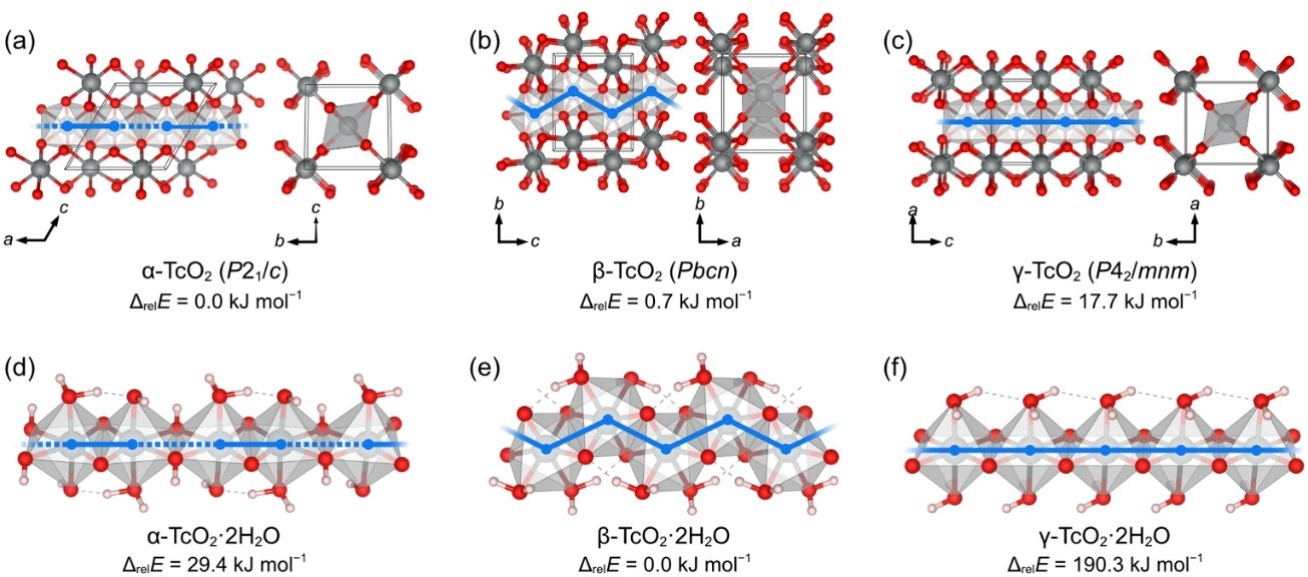
TcO_2_ crystal polymorphs and the TcO_2_ ⋅ 2H_2_O infinite chains derived therefrom. Tc atoms represented as octahedra emphasize the structure of laterally interconnected chains in the TcO_2_ crystals, and the primitive unit cells are indicated by black lines. The blue lines emphasize the linear and zigzag paths of the chains, with dashed lines indicating the longer Tc−Tc distances (in the case of alternating long and short distances). The TcO_2_ crystals and TcO_2_ ⋅ 2H_2_O chains were fully optimized with DFT. During the α‐TcO_2_ ⋅ 2H_2_O optimization, a few H atoms migrated from the H_2_O groups to μ‐O bridges. The relative energies per formula unit (Δ_rel_ *E*) of the optimized structures are shown.

The calculated TcO_2_ crystal structures are represented in Figure 1a–c. All three polymorphs consist of edge‐sharing TcO_6_ octahedra forming unidimensional chains that are laterally interconnected via corner‐sharing O atoms. The chain configuration is characteristic of each TcO_2_ polymorph: in α‐TcO_2_, the chains follow a straight path, with consecutive Tc−Tc pairs alternating between shorter and longer distances; the chains in γ‐TcO_2_ are also straight, but the Tc atoms are separated by identical distances; in β‐TcO_2_, consecutive Tc atoms are also separated by identical distances, but the chains follow a distinctive zigzag path. From each TcO_2_ crystal structure, we extracted a single chain and converted the terminal O atoms into H_2_O groups to balance the total electric charge, resulting in the TcO_2_ ⋅ 2H_2_O chains shown in Figure S2a‐c. We refer to these chains as α‐, β‐, and γ‐TcO_2_ ⋅ 2H_2_O, to indicate that the chains were derived from the α‐, β‐, and γ‐TcO_2_ crystal structures, respectively. We also considered hydroxide chains (Figure S2d–f) where all O atoms were converted into OH groups; however, these chains turned out to be energetically disfavored, as shown in Table S3.

All structures were fully optimized (lattice vectors and atomic coordinates) using AMS/BAND^[12]^ with the PBE^[13]^ density functional, scalar relativistic effects,^[14]^ and numerical atomic orbitals (NAOs) augmented with a triple‐zeta polarized (TZP) set of Slater‐type basis functions. For the chains, D3 dispersion corrections^[15]^ were also included. Further computational details are provided in the Experimental Section.

Figure 1d–f shows the optimized structures and relative energies calculated for the TcO_2_ ⋅ 2H_2_O chains. The β‐TcO_2_ ⋅ 2H_2_O structure (zigzag) is clearly the energetically most favored one; the energy difference to the next structure, α‐TcO_2_ ⋅ 2H_2_O, is already significantly high (29.4 kJ mol^−1^ per formula unit). Note that, whereas β‐ and γ‐TcO_2_ ⋅ 2H_2_O retained their general structure, α‐TcO_2_ ⋅ 2H_2_O rearranged into an oxyhydroxide chain, which can be represented as Tc(μ‐O)(μ‐OH)(OH)(H_2_O); nonetheless, for convenience, we will continue referring to this structure as α‐TcO_2_ ⋅ 2H_2_O.

**Figure 2:**
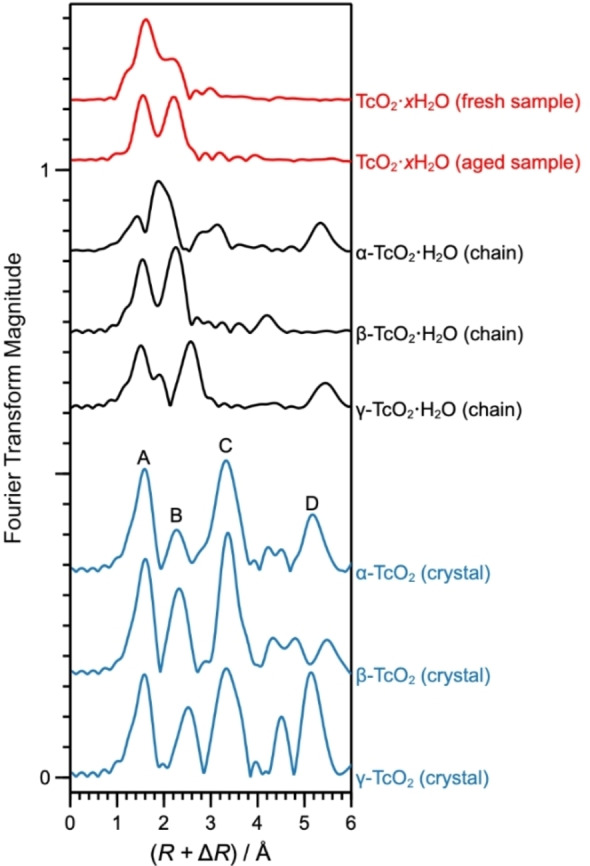
Fourier‐transform magnitudes (FTMs) from experimental EXAFS spectra of the fresh and aged TcO_2_ ⋅ *x*H_2_O precipitates (red curves) and from EXAFS spectra simulated with FEFF9.6.4^[17]^ for the TcO_2_ ⋅ H_2_O infinite chains (black curves) and TcO_2_ crystal structures (blue curves) optimized with DFT. The fresh sample was measured within a month of preparation;^[3a]^ the aged sample was stored for four years under room conditions prior to measurement. The geometry of α‐TcO_2_ ⋅ H_2_O converged to a mixed protonation state, as shown in Figure 1d). For the TcO_2_ crystals, peak A is associated with the first Tc−O coordination shell, B with the nearest intrachain Tc−Tc neighbors, C with the nearest interchain Tc−Tc neighbors, and D with the second intrachain Tc−Tc neighbors.

The Tc−O bond lengths as well as Tc−Tc distances calculated for the optimized TcO_2_ ⋅ 2H_2_O chains are shown in Table 1 in comparison with the corresponding parameters obtained for the TcO_2_ crystal structures and from EXAFS spectra. The EXAFS parameters were determined by shell fitting (for details see the Supporting Information) from experimental spectra of a *fresh* and an *aged* sample. The spectrum of the fresh sample is the one published by Yalçintaş et al.,^[3a]^ measured within a month after sample preparation; the shell fitting was redone in this work. The spectrum of the aged sample, on the other hand, is presented in this work for the first time; in this case, the TcO_2_ ⋅ *x*H_2_O precipitate was stored in air, at room temperature, for about four years prior to the EXAFS measurement. The experimental EXAFS spectra of both samples were obtained at the Rossendorf Beamline at ESRF,^[16]^ under identical conditions, most importantly, by using a closed‐cycle He cryostat to maintain a temperature of 15 K and anoxic conditions during the measurement. They are shown in Figure 2, in comparison with the simulated spectra of the TcO_2_ ⋅ 2H_2_O chains and TcO_2_ crystals calculated with DFT.

**Table 1:** Tc coordination numbers (CN) and interatomic distances (*R*) from EXAFS shell fitting of fresh and aged TcO_2_ ⋅ *x*H_2_O samples along with the values obtained from DFT for the TcO_2_ ⋅ 2H_2_O chains and TcO_2_ crystals. The CN values for the Tc−Tc interchain distances consider only one neighboring chain.

	Exptl. EXAFS (shell fitting)				DFT calculations											
	TcO_2_ ⋅ *x*H_2_O precipitates				Infinite chains						Crystal structures					
	Fresh^[a]^		Aged^[b]^		α‐TcO_2_ ⋅ 2H_2_O^[c]^		β‐TcO_2_ ⋅ 2H_2_O		γ‐TcO_2_ ⋅ 2H_2_O		α‐TcO_2_		β‐TcO_2_		γ‐TcO_2_	
Shell	CN	R/Å	CN	R/Å	CN	R/Å	CN	R/Å	CN	R/Å	CN	R/Å	CN	R/Å	CN	R/Å
Tc−(μ‐O)	4^[f]^	2.01	4^[f]^	2.01	2 2	1.93 2.11^[d]^	4	1.98	4	1.97	6	2.01	6	2.01	6	2.00
Tc−OH_2_	2^[f]^	2.39	2^[f]^	2.14	1 1	2.04^[e]^ 2.17	2	2.23	2	2.24	–	–	–	–	–	–
Tc−Tc (intrachain)	2^[f]^	2.55	2^[f]^ 0.6 4.2	2.54 4.63 7.03	1 1 2	2.34 3.38 5.71	2 2 2	2.53 4.60 6.98	2 2 2	2.90 5.80 8.70	1 1 2	2.62 3.12 5.73	2 2 2	2.64 4.71 7.16	2 2 2	2.84 5.68 8.53
Tc−Tc (interchain)	–	–	0.8 1.2 2.5	3.80 5.06 6.04	–	–	–	–	–	–	2 1 1	3.68 5.24 5.44	2 2 2	3.72 5.26 6.00	2 2 2	3.68 5.46 7.88

Errors of ±25 % are generally associated to CN values from EXAFS fitting. [a] Sample measured within a month from preparation.^[3a]^ [b] Sample measured after four years of storage at room conditions. [c] Structure converged to an oxyhydroxide chain (Figure 1d). [d] OH bridge. [e] Terminal OH group. [f] Fixed during fitting.

Table 1 clearly shows that, while the Tc−O bond lengths are characteristic of the chemical groups attached to the Tc atoms (μ‐O, O, μ‐OH, OH, and H_2_O), the Tc−Tc distances have characteristic values for each system, even if only the nearest Tc−Tc neighbors are considered. Thus, these systems would be clearly identifiable by EXAFS measurements. Note that the TcO_2_ ⋅ 2H_2_O chains keep the same general characteristics of the TcO_2_ crystal structures, that is, α‐ and γ‐TcO_2_ ⋅ 2H_2_O form a straight chain, with alternating shorter and longer Tc‐Tc distances in the former and only one Tc‐Tc distance in the latter. Similarly, β‐TcO_2_ ⋅ 2H_2_O – the energetically favored structure – remains as a zigzag chain with practically identical Tc−Tc distances.

The EXAFS Tc−O and Tc−Tc nearest‐neighbor distances from the fresh and aged samples are in very good agreement with the distances in the calculated β‐TcO_2_ ⋅ 2H_2_O chain, as shown in Table 1. The Tc‐Tc distances (which characterize the chain structure) differ by ≤0.02 Å, whereas the Tc−(μ‐O) bonds differ by ≤0.03 Å; the differences are slightly larger for the Tc−OH_2_ bonds (0.16 Å for the fresh sample and 0.09 Å for the aged sample), but still in good agreement in both cases. The discrepancy between Tc−OH_2_ bonds in the fresh and aged samples is reflected in the sharper splitting of the Fourier transform magnitude (FTM) peaks corresponding to the nearest Tc−O and Tc−Tc distances (peaks A and B in Figure 2). The most significant difference between the fresh and aged samples, however, is the absence of signals related to the longer Tc‐Tc distances in the former, either because of a high static disorder of the chains or because the chains are too short to show such backscattering pairs consistently (note that thermal disorder can be excluded since both the fresh and the aged samples were measured at 15 K).

**Figure 3:**
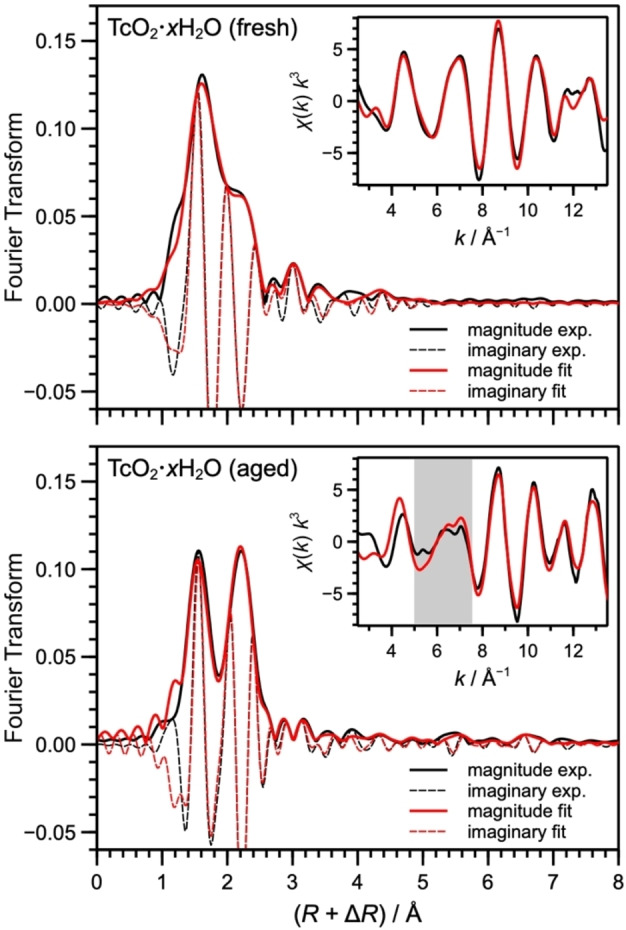
Experimental EXAFS spectra of fresh (top) and aged (bottom) TcO_2_ ⋅ *x*H_2_O samples (black traces) and their reproduction by shell fitting (red traces). The main plots show the Fourier transform magnitude (bold lines) and the imaginary part (thin lines); the inserts show the corresponding *k* ^3^‐weighted *χ* spectra (the shadowed region indicates high‐frequency signals absent in the fresh sample). The shell fit of the fresh sample is based on the DFT‐derived structure of β‐TcO_2_ ⋅ H_2_O chain, the shell fit of the aged sample is based on the DFT‐derived structure of the β‐TcO_2_ crystal. The fitted parameters are compiled in Tables 1 and S4.

**Figure 4:**
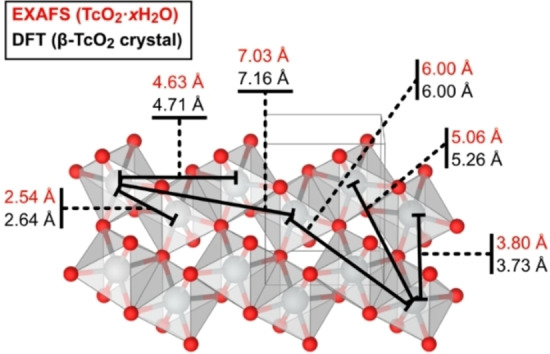
Intra‐ and interchain Tc−Tc distances. The DFT values (black) correspond to the optimized β‐TcO_2_ crystal structure, whereas the EXAFS values (red) were obtained from fitting the experimental spectrum of the aged TcO_2_ ⋅ *x*H_2_O sample.

The most conclusive proof of the formation of zigzag chains in the TcO_2_ ⋅ *x*H_2_O precipitates comes from the EXAFS analysis of the aged sample. As shown in Figure 3, the *χ*(*k*) spectrum of the aged precipitate contains high‐frequency signals between 5 and 7 Å^−1^ that are absent in the spectrum of the fresh sample. These signals correspond to second and third intrachain Tc−Tc distances of 4.63 and 7.03 Å shown in Table 1, which are only possible in the β‐TcO_2_ ⋅ 2H_2_O chain (calculated as 4.71 and 7.16 Å). Interestingly, Tc−Tc distances of 3.80, 5.06, and 6.04 Å, corresponding to the interchain distances observed in the β‐TcO_2_ crystal, could also be fitted, indicating that the precipitate develops a tridimensional organization with aging (Figure 4). Nonetheless, in the sample analyzed here, this tridimensional structure is likely in its initial stage, otherwise the EXAFS spectrum would bear a stronger resemblance to the spectrum of the β‐TcO_2_ crystal in Figure 2, especially with respect to peak C.

The absence of interpretable EXAFS signals related to longer Tc−Tc distances in the fresh sample and the presence of the signals associated with long, interconnected parallel chains in the aged sample is indicative of the aging process in TcO_2_ ⋅ *x*H_2_O precipitates. In our interpretation, the short β‐TcO_2_ ⋅ 2H_2_O chains combine with each other to form longer zigzag chains via condensation reactions, thereby releasing H_2_O. As the chains become longer, condensation reactions leading to cross‐linked chains start to take place. The shortening of the second Tc‐O path in the experimental EXAFS (denoted Tc−OH_2_ in Table 1) from 2.39 Å in the fresh to 2.14 Å in the aged sample is an additional indication of this condensation process. This scenario is consistent with the thermodynamic evidence compiled by Grambow^[18]^ that TcO_2_ ⋅ *x*H_2_O would release H_2_O over time and that the process takes place very slowly; as discussed above, after four years the aged sample still shows early signs of cross‐linking between chains.

## Conclusion

In conclusion, by combining DFT calculations with EXAFS measurements, we have demonstrated that, contrary to the currently anticipated linear structure, TcO_2_ ⋅ *x*H_2_O precipitates are polymeric structures formed of zigzag chains of edge‐sharing TcO_6_ octahedra with terminal H_2_O ligands at the *cis* positions (Figure 1e). Differences in the EXAFS of a fresh sample and a sample aged for four years indicate that the length of the polymeric chains increases slowly over time and might lead to crystallization of a yet uncharacterized TcO_2_ phase analogous to β‐ReO_2_ (Figure 1b), which our calculations (Table S2) show to be energetically equivalent to the known *P*2_1_/*c* ^[10]^ (α‐TcO_2_) phase.

## Experimental Section

### DFT calculations

*TcO_2_ and ReO_2_ crystal structures*: The initial TcO_2_ and ReO_2_ structures were constructed from crystallographic data for α‐ReO_2_ ^[7]^ (monoclinic, *P*2_1_/*c*), β‐ReO_2_ ^[8]^ (orthorhombic, *Pbcn*), and γ‐ReO_2_ ^[9]^ (tetragonal, *P*4_2_/*mnm*), depicted in Figure S1. The equivalent TcO_2_ structures were obtained simply by replacing the Re atoms with Tc. Finally, the tetragonal phases (γ‐TcO_2_ and γ‐ReO_2_) were duplicated in the *c* direction (1×1×2 supercells), so that all cells would contain four TcO_2_ or ReO_2_ formula units. Subsequently, the atomic coordinates and lattice vectors were fully optimized using the PBE^[13]^ density functional with numerical atomic orbitals (NAOs) augmented with a triple‐zeta polarized (TZP) set of Slater‐type basis functions; relativistic effects were included by using the zeroth‐order regular approximation (ZORA).^[14]^ These calculations were conducted with the AMS/BAND^[12]^ program, using regular *k*‐space grids with quality set to “Good” and default convergence criteria.

*TcO_2_ ⋅ 2H_2_O infinite chains*: The initial TcO_2_ ⋅ 2H_2_O chains were constructed by extracting a single chain of edge‐sharing TcO_6_ octahedra from each TcO_2_ crystal phase and saturating the O atoms with H according to two approaches, as shown in Figure S2: (a−c) terminal O atoms were converted into H_2_O ligands and bridging O atoms were left unprotonated, resulting in (Tc(μ‐O)_2_(OH_2_))_*n*_ chains; (d−f) terminal and bridging O atoms were converted into OH groups, resulting in (Tc(OH)_2_(μ‐OH)_2_)_*n*_ chains. Finally, the atomic coordinates and lattice parameter of each structure were fully optimized using the method described above for the crystal structures, supplemented with Grimme's D3 corrections^[15]^ to improve the description of dispersion interactions with OH and H_2_O groups. Like for the crystal structures, these calculations were conducted with the AMS/BAND^[12]^ program, using regular *k*‐space grids with quality set to “Good”. Note that these systems are periodic only along the chain direction.

*Single‐point calculations*: Energies and electronic properties were calculated for the optimized structures with the same methods used in the preceding geometry optimizations, except for a denser *k*‐space grid (quality set to “VeryGood”). For comparison, single‐point calculations were also carried out within the FHI‐aims program,^[19]^ using the PBE^[13]^ and HSE06^[20]^ density functionals with a “tier 1” set of atom‐centered NAO basis functions; relativistic effects were described with the “atomic ZORA” approach;^[19]^ for the TcO_2_ ⋅ 2H_2_O infinite chains, the Tkatchenko‐Scheffler (TS) method^[21]^ was used for the dispersion interactions.

### TcO_2_ ⋅ xH_2_O samples and EXAFS measurements

*Fresh sample*: The preparation and EXAFS spectrum of the fresh sample were reported by Yalçintaş et al.^[3a]^ The TcO_2_ ⋅ *x*H_2_O precipitate was prepared by acidifying a pertechnetate solution with concentrated HCl, then adding Zn to generate nascent hydrogen. After the reaction was completed, NaOH (20 M) was added to obtain a black precipitate, which aged for one week. The sample was stored under Ar at liquid nitrogen temperature. The EXAFS was measured within 30 days of preparation at the Rossendorf Beamline (BM20 at ESRF, Grenoble, France) in fluorescence mode at the Tc K‐edge (21044 eV). The sample was kept at 15 K during the measurement. Further details can be found in the original publication.^[3a]^

*Aged sample*: The sample was prepared by hydrolysis of K_2_[TcCl_6_], which was previously synthesized by an established procedure.^[22]^ Solid K_2_[TcCl_6_] (390 mg, 1 mmol) was dissolved in 0.5 mL H_2_O, which resulted in the precipitation of a dark brown solid. An aqueous solution of KOH (2 mL, 0.1 M) was added, and the suspension was stirred for 5 h at room temperature. The formed black brown solid was filtered off and washed with water (5×1 mL). The absence of Cl^−^ in the final washing solution was checked by the addition of Ag(NO_3_). The thus formed TcO_2_ ⋅ *x*H_2_O was dried in air at room temperature. Yield: practically quantitative. The sample was prepared and stored at room temperature in air for four years prior to the EXAFS measurement, which was conducted identically to the fresh sample.

### EXAFS analyses

*EXAFS shell fitting*: Tc K‐edge EXAFS shell fittings were conducted in R‐space with WinXAS^[23]^ using the β‐TcO_2_ ⋅ 2H_2_O chain structure optimized with DFT (Figure 2e) for the oxygen coordination to Tc and for intrachain Tc−Tc distances. Interchain Tc−Tc distances from the DFT‐derived β‐TcO_2_ crystal structure were also included. The spectrum of the fresh precipitate was well fit by four oxygen atoms at 2.01 Å and two oxygen atoms at 2.39 Å, and 8 additional four‐legged multiple scattering paths arising from the quasi square‐planar configuration of the four nearest oxygen atoms (Table S4). Furthermore, we obtained two Tc atoms at 2.55 Å. These radial distances are in excellent agreement with the local structure of β‐TcO_2_ ⋅ 2H_2_O, except for the two longer Tc−O distances, which represent the two water molecules in the coordination sphere and are about 0.2 Å longer than predicted by DFT. Note that a similarly long distance has been determined by Lukens et al.,^[4]^ also by EXAFS shell fitting. The spectrum of the fresh precipitate does not reveal any backscattering peaks at longer Tc−Tc distances that could be fitted. For the aged sample, in addition to the local structure obtained for the fresh sample, Tc−Tc distances corresponding to the 2nd and 3rd intrachain shells and for the three interchain shells (Figure 4) could also be fitted (Table S4 and Figure 3), in good agreement with the parameters in the β‐TcO_2_ ⋅ 2H_2_O chain and β‐TcO_2_ crystal. The amplitude reduction factor *S*_0_^2^ was fixed to 0.8 for all fits.

*EXAFS simulation*: EXAFS spectra were simulated for the DFT‐optimized structures with the program FEFF9.6.4^[17]^ using the self‐consistent field mode with a global Debye‐Waller factor of 0.003 Å, amplitude reduction factor of 0.9, and Δ*E*_0_=0.

## Conflict of interest

The authors declare no conflict of interest.

1

## Supporting information

As a service to our authors and readers, this journal provides supporting information supplied by the authors. Such materials are peer reviewed and may be re‐organized for online delivery, but are not copy‐edited or typeset. Technical support issues arising from supporting information (other than missing files) should be addressed to the authors.

Supporting Information

## Data Availability

The data that support the findings of this study are available in the supplementary material of this article.

## References

[1] R. Alberto , COSMOS 2012, 8, 83.

[2] J. P. Icenhower , N. P. Qafoku , J. M. Zachara , W. J. Martin , Am. J. Sci. 2010, 310, 721.

[3a] E. Yalçıntaş , A. C. Scheinost , X. Gaona , M. Altmaier , Dalton Trans. 2016, 45, 17874; 27775147 10.1039/c6dt02872a

[3b] K. Morris , F. R. Livens , J. M. Charnock , I. T. Burke , J. M. McBeth , J. D. C. Begg , C. Boothman , J. R. Lloyd , Appl. Geochem. 2008, 23, 603;

[3c] T. S. Peretyazhko , J. M. Zachara , R. K. Kukkadapu , S. M. Heald , I. V. Kutnyakov , C. T. Resch , B. W. Arey , C. M. Wang , L. Kovarik , J. L. Phillips , D. A. Moore , Geochim. Cosmochim. Acta 2012, 92, 48;

[3d] J. M. McBeth , J. R. Lloyd , G. T. W. Law , F. R. Livens , I. T. Burke , K. Morris , Mineral. Mag. 2011, 75, 2419.

[4] W. W. Lukens , J. J. Bucher , N. M. Edelstein , D. K. Shuh , Environ. Sci. Technol. 2002, 36, 1124. 11918000 10.1021/es015653+

[5] L. Vichot , G. Ouvrard , G. Montavon , M. Fattahi , C. Musikas , B. Grambow , Radiochim. Acta 2002, 90, 575.

[6] I. Almahamid , J. C. Bryan , J. J. Bucher , A. K. Burrell , N. M. Edelstein , E. A. Hudson , N. Kaltsoyannis , W. W. Lukens , D. K. Shuh , H. Nitsche , T. Reich , Inorg. Chem. 1995, 34, 193.

[7] F. F. Ferreira , H. P. S. Correa , M. T. D. Orlando , J. L. Passamai Jr. , C. G. P. Orlando , I. P. Cavalcante , F. Garcia , E. Tamura , L. G. Martinez , J. L. Rossi , F. C. L. de Melo , J. Synchrotron Radiat. 2009, 16, 48. 19096174 10.1107/S0909049508036029

[8] A. Magnéli , Acta Chem. Scand. 1957, 11, 28.

[9] A. L. Ivanovskii , T. I. Chupakhina , V. G. Zubkov , A. P. Tyutyunnik , V. N. Krasilnikov , G. V. Bazuev , S. V. Okatov , A. I. Lichtenstein , Phys. Lett. A 2005, 348, 66.

[10] E. E. Rodriguez , F. Poineau , A. Llobet , A. P. Sattelberger , J. Bhattacharjee , U. V. Waghmare , T. Hartmann , A. K. Cheetham , J. Am. Chem. Soc. 2007, 129, 10244. 17655304 10.1021/ja0727363

[11a] S. Duckworth , X. Gaona , D. Castaño , S. Park , M. Altmaier , H. Geckeis , Appl. Geochem. 2021, 105037;

[11b] P. V. Grundler , L. Helm , R. Alberto , A. E. Merbach , Inorg. Chem. 2006, 45, 10378. 17140248 10.1021/ic061578y

[12a] G. te Velde , E. J. Baerends , Phys. Rev. B 1991, 44, 7888; 10.1103/physrevb.44.7888 9998719

[12b] P. H. T. Philipsen, G. t. Velde, E. J. Baerends, J. A. Berger, P. L. d. Boeij, M. Franchini, J. A. Groeneveld, E. S. Kadantsev, R. Klooster, F. Kootstra, P. Romaniello, M. Raupach, D. G. Skachkov, J. G. Snijders, C. J. O. Verzijl, J. A. C. Gil, J. M. Thijssen, G. Wiesenekker, C. A. Peeples, G. Schreckenbach, T. Ziegler, BAND2019, SCM, Theoretical Chemistry, Vrije Universiteit, Amsterdam, The Netherlands, **2019**;

[12c] R. Rüger, M. Franchini, T. Trnka, A. Yakovlev, E. v. Lenthe, P. Philipsen, B. Klumpers, T. Soini, AMS 2019, SCM, Theoretical Chemistry, Vrije Universiteit, Amsterdam, The Netherlands, **2019**.

[13] J. P. Perdew , K. Burke , M. Ernzerhof , Phys. Rev. Lett. 1996, 77, 3865. 10062328 10.1103/PhysRevLett.77.3865

[14] P. H. T. Philipsen , E. vanLenthe , J. G. Snijders , E. J. Baerends , Phys. Rev. B 1997, 56, 13556.

[15] S. Grimme , J. Antony , S. Ehrlich , H. Krieg , J. Chem. Phys. 2010, 132, 154104. 20423165 10.1063/1.3382344

[16] A. C. Scheinost , J. Claussner , J. Exner , M. Feig , S. Findeisen , C. Hennig , K. O. Kvashnina , D. Naudet , D. Prieur , A. Rossberg , M. Schmidt , C. Qiu , P. Colomp , C. Cohen , E. Dettona , V. Dyadkin , T. Stumpf , J. Synchrotron Radiat. 2021, 28, 333. 33399586 10.1107/S1600577520014265 PMC7842221

[17a] J. J. Rehr , R. C. Albers , Rev. Mod. Phys. 2000, 72, 621;

[17b] J. J. Rehr , J. J. Kas , M. P. Prange , A. P. Sorini , Y. Takimoto , F. Vila , C. R. Phys. 2009, 10, 548;

[17c] J. J. Rehr , J. J. Kas , F. D. Vila , M. P. Prange , K. Jorissen , Phys. Chem. Chem. Phys. 2010, 12, 5503. 20445945 10.1039/b926434e

[18] I. Grenthe, X. Gaona, L. Rao, A. Plyasunov, W. Runde, B. Grambow, R. Konings, A. Smith, E. Moore, M.-E. Ragoussi, J. S. Martinez, D. Costa, A. Felmy, K. Spahiu, *Second Update on the Chemical Thermodynamics of Uranium, Neptunium, Plutonium, Americium and Technetium, Vol. 14*, Nuclear Energy Agency of the OECD (NEA), **2021**.

[19] V. Blum , R. Gehrke , F. Hanke , P. Havu , V. Havu , X. Ren , K. Reuter , M. Scheffler , Comput. Phys. Commun. 2009, 180, 2175.

[20a] J. Heyd , G. E. Scuseria , M. Ernzerhof , J. Chem. Phys. 2003, 118, 8207;

[20b] J. Heyd , G. E. Scuseria , M. Ernzerhof , J. Chem. Phys. 2006, 124, 219906;

[20c] A. V. Krukau , O. A. Vydrov , A. F. Izmaylov , G. E. Scuseria , J. Chem. Phys. 2006, 125, 224106. 17176133 10.1063/1.2404663

[21] A. Tkatchenko , M. Scheffler , Phys. Rev. Lett. 2008, 102, 073005. 10.1103/PhysRevLett.102.073005 19257665

[22] K. Schwochau in Handbuch der Präparativen Anorganischen Chemie, Vol. 3 (Ed.: G. Brauer ), Stuttgart, 1981, pp. 1597.

[23] T. Ressler , J. Synchrotron Radiat. 1998, 5, 118. 16687813 10.1107/S0909049597019298

