# Supplementary material for: Shedding Light on the Enigmatic TcO2 ⋅ xH2O Structure with Density Functional Theory and EXAFS Spectroscopy
Source: Chemistry. 2022 Sep 23;28(59):e202202235. doi: 10.1002/chem.202202235 (PMC9826482; doi:10.1002/chem.202202235)
Supplement: Supplementary file 1 — Supporting Information [file CHEM-28-0-s001.pdf]

Chemistry–A European Journal

Supporting Information

Shedding Light on the Enigmatic $TcO_2 \cdot xH_2O$ Structure with Density Functional Theory and EXAFS Spectroscopy

Augusto F. Oliveira,* Agnieszka Kuc, Thomas Heine, Ulrich Abram, and Andreas C. Scheinost*

Table of Contents

Table of Contents 1
1. Additional Results and Discussion 2
1.1. TcO_2 and ReO_2 Crystal Structures 2
1.2. $TcO_2 \cdot 2H_2O$ Chain Structures 2
1.3. EXAFS Shell Fitting 3
References 10

1. Additional Results and Discussion

1.1. TcO_2 and ReO_2 Crystal Structures

Table S1 shows the optimized lattice parameters and nearest-neighbor distances for the TcO_2 and ReO_2 crystals in comparison with experimental values (except for β- and γ-TcO_2, which have not been characterized experimentally to date). The calculated cell volumes are slightly overestimated, between 2.3% (γ-ReO_2) and 3.5% (α-TcO_2), with an average of ca. 3%. The lattice vectors deviate from -1.9% to 2.1%, averaging at 1%, whereas differences in the lattice angles are negligible. The M-O nearest-neighbor distances (M = Tc, Re) are very similar among all crystals, fluctuating between 1.98 Å and 2.02 Å. The M-M distances, in contrast, are very characteristic of the crystal systems and are also in good agreement with experimental values, with deviations within ±0.06 Å in most cases. The largest deviation was obtained for the longer Re-Re distance in α-ReO_2: 0.14 Å with respect to the XRD structure by Ferreira et al.[1]; nevertheless, we note that this difference gives an error of less than 5% and that the EXAFS results reported in the same publication show similar deviations with respect to the XRD structure.

Although the crystallographic structures of β- and γ-TcO_2 have not been characterized experimentally, the geometry optimizations converged to structures very similar to their ReO_2 analogues (see Table S1). The largest difference arises for the M-M distance in the tetragonal phases, which is ca. 0.1 Å shorter in γ-ReO_2 than in γ-TcO_2. Thus, the calculations show that the β- and γ-TcO_2 structures indeed correspond to local minima on the potential energy surface of TcO_2 and might be possible to be synthesized.

Table S2 shows the relative energies calculated for the optimized TcO_2 and ReO_2 crystals with the DFT methods described above. For ReO_2, despite differences in the absolute values, all methods show the orthorhombic structure (β-ReO_2) as the most energetically favored phase, followed by α- and γ-ReO_2, respectively. This is consistent with the fact that α-ReO_2 converts irreversibly into β-ReO_2 when heated above 300 °C[2] and that epitaxial growth of ReO_2 films on TiO_2 (001) transitions from γ- to α-ReO_2 at a thickness of ca. 25 nm of height, where the lattice strain induced by the TiO_2 substrate becomes less effective.[3] For TcO_2, while the γ phase is also the least favored energetically, α- and β-TcO_2 can be considered energetically equivalent within the expected DFT accuracy, as evidenced by the inversion of the results between AMS/BAND and FHI-aims. This is rather surprising since β-TcO_2 has not been observed experimentally and α-TcO_2 has been shown to be thermally stable in temperatures up to at least 1273 K.[4]

Figure S3 shows the electronic density of states (DOS) calculated for the TcO_2 and ReO_2 crystals with different methods. The DOS obtained with PBE in AMS/BAND and FHI-aims are almost identical (as expected), but differ from the HSE06 results; however, these are not qualitative differences, i.e., they correspond to a linear scaling of the energy values that result in bandwidths ca. 14% wider for HSE06 in comparison to PBE. Thus, we only analyze the projected DOS for the PBE calculations done with AMS/BAND.

Figure S4 shows the DOS projections on the TcO_2 and ReO_2 valence orbitals and the crystal orbital overlap populations (COOPs) for the M-M and M-O interactions (M = Tc, Re). All six phases have metallic character, with states crossing the Fermi level. Three regions can be identified in the DOS plots, from lower to higher energy: (i) a region dominated by O p states; (ii) a region that crosses the Fermi level, with prevalent M d character; (iii) a fully unoccupied region, also with predominantly M d character. Despite the dominant character in each region, the commensurate bandwidths of the O p and M d states indicate a covalent bonding between the metal and oxygen atoms, consistent with calculations found in the literature[5].

Stronger evidence of the covalent interactions is shown by the COOP plots in Figure S4. The plots confirm that first region of the DOS plots contains M d-O p bonding states (COOP > 0), whereas the second region contains M d-O p antibonding states (COOP < 0); overall, the M d-O p interactions have bonding character, indicated by the COOP integral up to the Fermi energy. The COOPs also reveal M d-M d covalent states in the second region, with a positive peak followed by a negative one; for the α and γ phases, the antibonding peak is centered at the Fermi level, practically cancelling the bonding character of the M-M overlap; but for the β-MO_2 phases, the antibonding M d-M d overlaps are located above the Fermi level, resulting in the highest M-M bonding character among the crystal phases, especially for M = Re. We note, however, that when considering both the M d-M d and M d-O p overlaps, the total bonding character is higher for the α- and γ-phases, which is in contradiction with the relative energies shown in Table S2; thus, it is likely that the covalent bonds are not sufficient to explain the relative stability of the crystal phases.

In summary, the calculation results for the TcO_2 and ReO_2 crystals validate the DFT method used in this work and demonstrate that it is suitable for the investigation of the $TcO_2 \cdot xH_2O$ structure. The calculations also show that β- and γ-TcO_2 (still unobserved experimentally) correspond to local minima in the TcO_2 potential energy surface and might eventually be synthesized, especially β-TcO_2, which turned out to be energetically equivalent to the experimentally characterized α-TcO_2.[5a]

1.2. $TcO_2 \cdot 2H_2O$ Chain Structures

Table S3 shows the relative energies calculated for the optimized $TcO_2 \cdot 2H_2O$ chains with different DFT methods. The values calculated with FHI-aims using the PBE-TS and HSE06-TS density functionals show the same trend of the PBE-D3 in AMS/BAND results discussed in the communication paper, even though the relative energies from HSE06-TS are smaller. We note that the

γ-$TcO_2 \cdot 2H_2O$ chain failed to converge with HSE06-TS; however, considering that PBE shows γ-$TcO_2 \cdot 2H_2O$ as the least energetically favored structure and the qualitative agreement between the HSE06-TS and PBE calculations, we have no reason to believe that the missing data would affect any of the conclusions presented in this work.

Structural parameters for the $TcO_2 \cdot 2H_2O$ chains optimized with PBE-D3 in AMS/BAND are shown in Table 1 and discussed in the main communication paper.

1.3. EXAFS Shell Fitting

The parameters obtained from the experimental EXFAS spectra of the fresh and aged samples are summarized in Table S4 and discussed in the main communication paper.

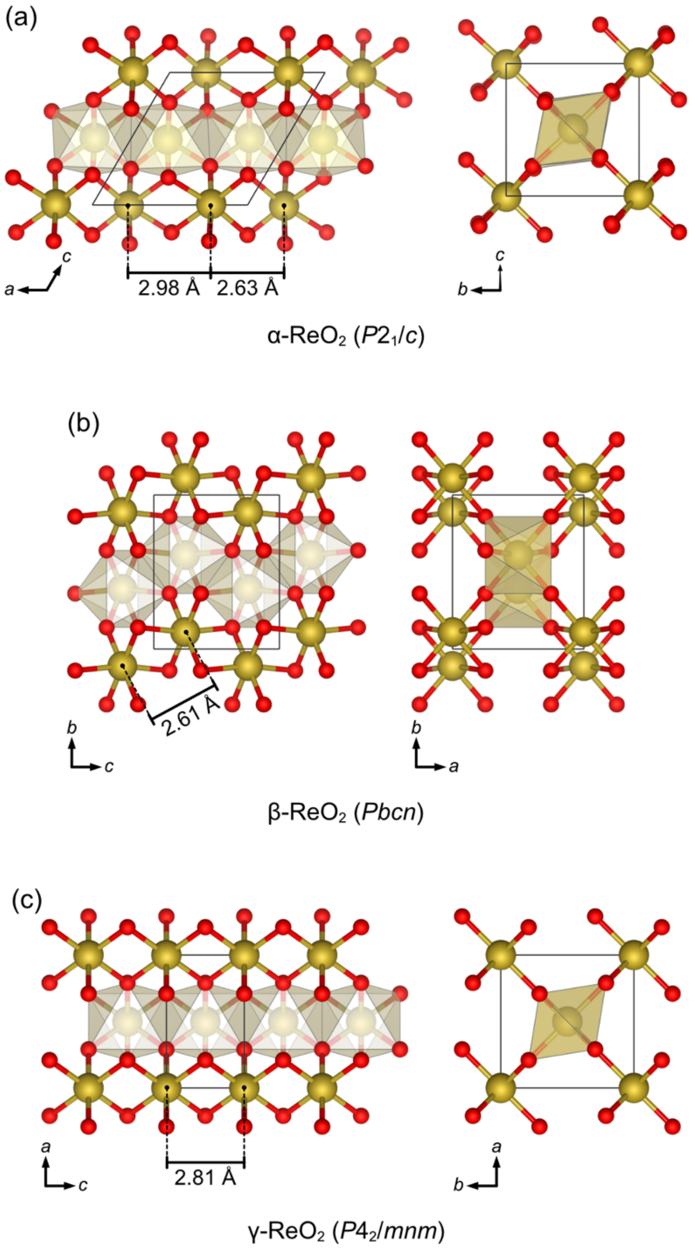

Figure S1. Crystal structure representation of the ReO_2 polymorphs. The structures are composed of laterally interconnected chains of edge-sharing ReO_6 octahedra. The ReO_6 units of the central chain are represented as polyhedrons in each model. Although α- and γ-ReO_2 are both formed of linear chains, neighboring Re atoms are separated by alternating longer and shorter distances along the chains in α-ReO_2, whereas all Re-Re distances are the same in γ-ReO_2. The primitive unit cells are indicated with black lines.

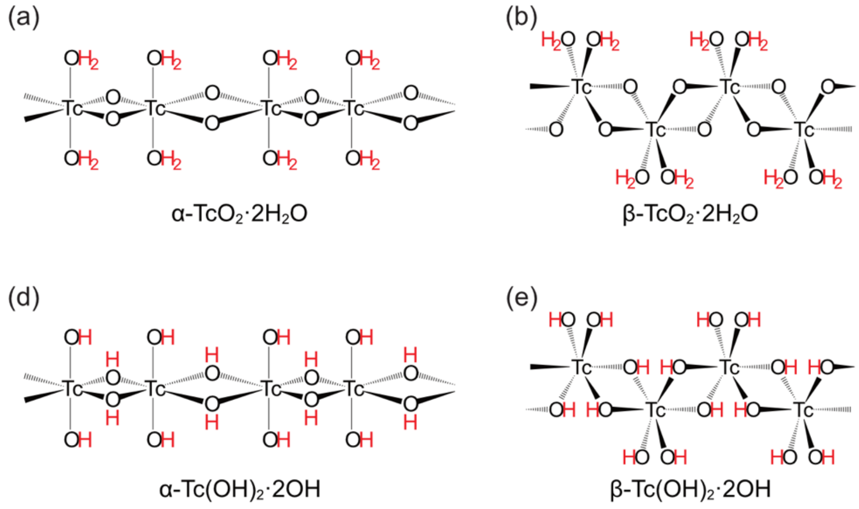

Figure S2. Representation of the initial $TcO_2 \cdot 2H_2O$ (a-c) and $Tc(OH)_2 \cdot 2OH$ (d-e)chain structures constructed from the optimized TcO_2 crystal structures. The $Tc(OH)_2 \cdot 2OH$ chains turned out to be energetically unfavored (see Table S3) and were not further analyzed. The optimized structures resulting from models a-c are show in Figure 1.

Table S1. Lattice constants and nearest interatomic distances for the TcO_2 and ReO_2 crystal structures. Selected values from literature are shown for comparison.

	a/Å	b/Å	c/Å	β/°	V_{cell}/Å^3	$R_{M\text{-}O}$/Å[a]	$R_{M\text{-}M}$/Å
**α-TcO_2**							
PBE (this work)	5.733	4.825	5.588	121.5	131.8	2.01(3)	2.62<br>3.12
LDA[5a][b]	5.689	4.755	5.519	121.5	127.4	1.98	2.59<br>3.11
NPD[5a]	5.689	4.755	5.519	121.5	127.4	1.98(2)	2.62<br>3.08
SXRD/NPD[4]	5.692	4.762	5.523	121.5	127.6	1.98(2)	2.61<br>3.09
SXRD/NPD (900 °C)[4]	5.756	4.775	5.561	121.5	130.3	1.98(2)	2.68<br>3.08
EXAFS[6]	—	—	—	—	—	1.98	2.61<br>3.10
**α-ReO_2**							
PBE (this work)	5.693	4.850	5.626	121.1	133.1	2.01(3)	2.58<br>3.12
XRD[1]	5.611	4.805	5.548	120.3	129.2	1.99(5)	2.63<br>2.98
EXAFS[1]	—	—	—	—	—	2.00	2.54<br>3.09
**β-TcO_2**							
PBE (this work)	4.760	5.708	4.706	90.0	127.9	2.01(1)	2.64
**β-ReO_2**							
PBE (this work)	4.799	5.746	4.650	90.0	128.2	2.02(0)	2.63
XRD[2]	4.809	5.643	4.601	90.0	124.9	2.00(8)	2.61
**γ-TcO_2[a]**							
PBE (this work)	4.813	4.813	5.684	90.0	131.6	2.00(3)	2.84
**γ-ReO_2[c]**							
PBE (this work)	4.900	4.900	5.509	90.0	132.3	2.01(4)	2.75
XRD/NPD[5b]	4.798	4.798	5.615	90.0	129.3	1.99(1)	2.81

[a] Average values; standard deviations (when available) indicated in parentheses. [b] Calculation performed using experimental values for the lattice parameters. [c] Values for 1×1×2 supercell.

Table S2. Relative energies in kJ mol^{-1} per formula unit for the TcO_2 and ReO_2 crystal structures.

	PBE	PBE (FHI-aims)[a]	HSE06 (FHI-aims)[a]
**TcO_2 crystal phases**			
α-TcO_2	0.0	4.3	1.8
β-TcO_2	0.7	0.0	0.0
γ-TcO_2	17.7	20.1	25.5
**ReO_2 crystal phases**			
α-ReO_2	20.0	22.1	28.8
β-ReO_2	0.0	0.0	0.0
γ-ReO_2	33.2	35.5	47.0

[a] Single-point calculations for the structures optimized with PBE.

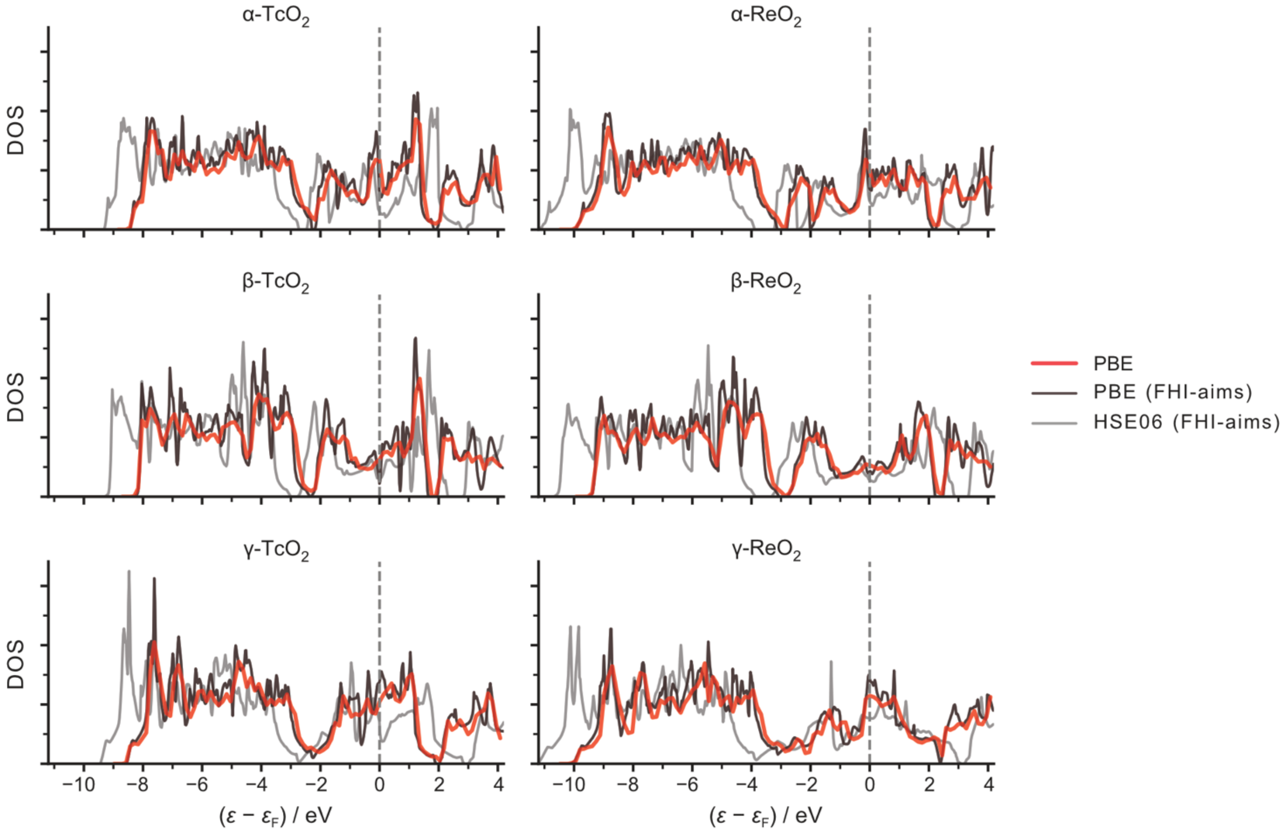

Figure S3. Electronic density of states (DOS) calculated with different methods for the TcO_2 and ReO_2 crystal structures optimized with PBE in AMS/BAND. The energies are shown with respect to the Fermi level, indicated by the vertical dashed lines. Differences between PBE in AMS/BAND and in FHI-aims are rather negligible. Despite differences in the energy values, HSE06 follows the same trends as PBE; in fact, an almost perfect match is obtained by scaling the HSE06 energies by a factor of ca. 0.87.

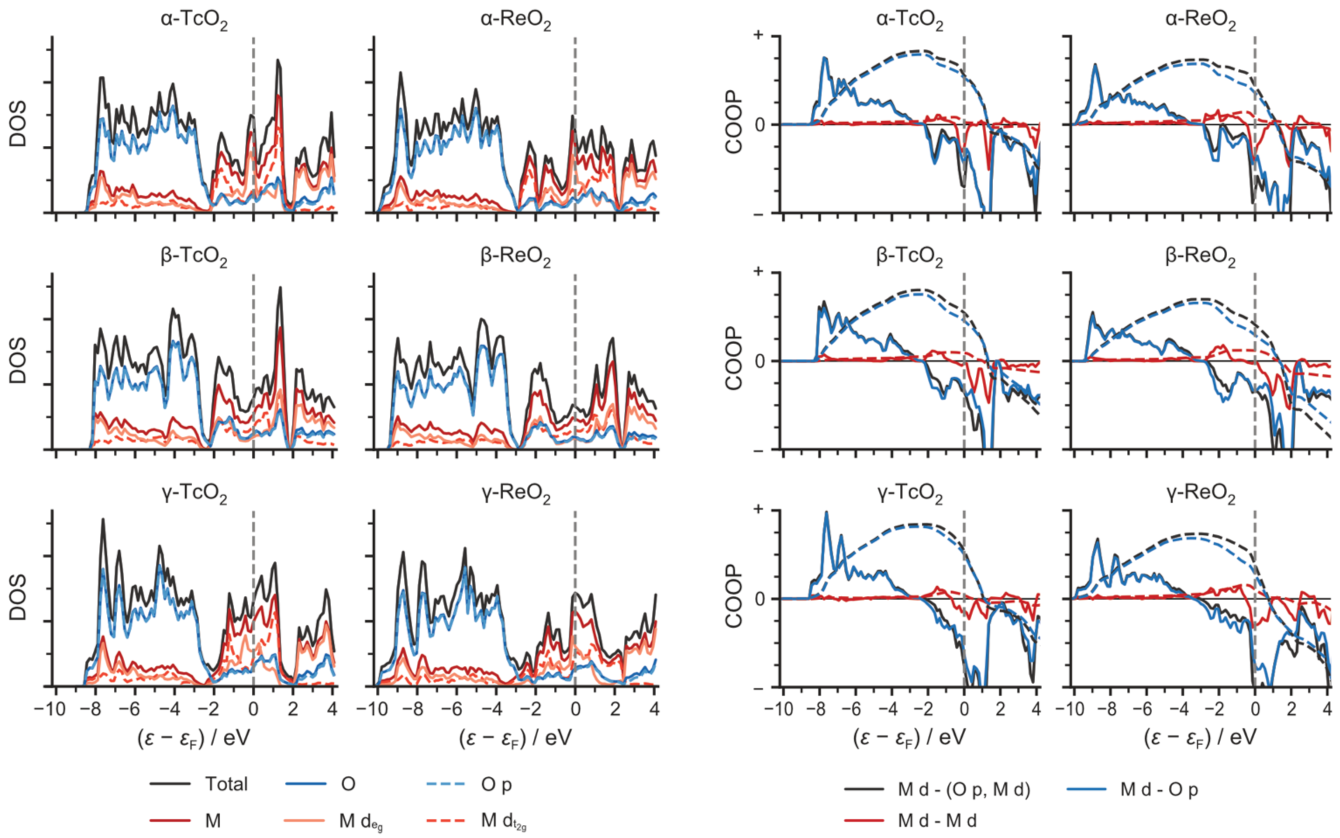

Figure S4. Electronic density of states (DOS, left side) and crystal orbital overlap population (COOP, right side) calculated with PBE density function on AMS/BAND for the optimized TcO_2 and ReO_2 crystal structures. Energies are shown with respect to the Fermi level (indicated by vertical dashed lines). In COOP, positive and negative values indicate bonding and antibonding orbital overlap, respectively; the corresponding cumulative COOP integrals are shown as dashed lines. The COOP integral over the occupied states (i.e., up to the Fermi level) correlates to the bonding order of the system.

Table S3. Selected interatomic distances optimized with the PBE-D3 DFT functional and relative energies calculated for the $TcO_2 \cdot 2H_2O$ infinite chains with different DFT functionals using the PBE-D3 geometry.

	Interatomic distances (PBE-D3)			Relative energies (in kJ mol^{-1} per formula unit)		
	Shell	CN	R/Å	PBE-D3	PBE-TS (FHI-aims)[a]	HSE06-TS (FHI-aims)[a]
α-$TcO_2 \cdot 2H_2O$[b]	Tc-(μ-O)	2<br>2	1.93<br>2.11[d]	29.4	23,4	13.9
	Tc-OH_2	1<br>1	2.04[e]<br>2.17			
	Tc-Tc	1<br>1	2.34<br>3.38			
β-$TcO_2 \cdot 2H_2O$	Tc-(μ-O)	4	1.98	0.0	0.0	0.0
	Tc-OH_2	2	2.23			
	Tc-Tc	2	2.53			
γ-$TcO_2 \cdot 2H_2O$	Tc-(μ-O)	4	1.97	190.3	189.5	—[f]
	Tc-OH_2	2	2.24			
	Tc-Tc	2	2.90			
α-$Tc(OH)_2 \cdot 2OH$	Tc-(μ-OH)	4	2.09	73.3	75.7	55.1
	Tc-OH	2	1.97			
	Tc-Tc	1<br>1	2.43<br>3.23			
β-$Tc(OH)_2 \cdot 2OH$[c]	Tc-(μ-OH)	4	2.13	79.3	78.7	42.6
	Tc-OH	2	1.91			
	Tc-Tc	1<br>1	2.53<br>3.39			
γ-$Tc(OH)_2 \cdot 2OH$	Tc-(μ-OH)	4	2.09	95.9	103.7	87.5
	Tc-OH	2	1.95			
	Tc-Tc	2	2.73			

[a] Single-point calculations for structures optimized with PBE-D3. [b] Geometry optimization converges to a hybrid protonation state with one H transferring from one H_2O group of each Tc to a neighboring μ-O bridge. [c] Final geometry is a zigzag chain with alternating shorter and longer Tc-Tc distances. [d] OH bridge. [e] Terminal OH group. [f] SCF did not converge.

Table S4. Parameters from EXAFS shell fitting for the fresh and aged $TcO_2 \cdot xH_2O$ samples.

	Fresh sample[a]			Aged sample[b]		
Shell	CN	R/Å	σ^2/Å^2	CN	R/Å	σ^2/Å^2
Tc-(μ-O)	4[f]	2.01	0.0020	4[f]	2.01	0.0010
Tc-OH_2	2[f]	2.39	0.0022	2[f]	2.14	0.0100
Tc-O-Tc-O	8[f]	4.02	0.0100	8[f]	4.00	0.0100
Tc-Tc (intrachain)	2[f]	2.55	0.0076	2[f]<br>0.6<br>4.2	2.54<br>4.63<br>7.03	0.0026<br>0.0028<br>0.0072
Tc-Tc (interchain)	—	—	—	0.8<br>1.2<br>2.5	3.80<br>5.06<br>6.04	0.0100<br>0.0100<br>0.0072
ΔE_0/eV[c]	-8.2			-13.8		
χ_{res}/%[d]	6.1			11.8		

The amplitude reduction factor S_0^2 was fixed at 0.8 for both samples. Errors of ±25% are generally associated to CN values from EXAFS fittings. [a] Spectrum acquired within one month from sample preparation. [b] Sample stored in room conditions for four years prior to EXAFS measurements. [c] Phase-shift. [d] Fitting residual. [f] Fixed value.

References

[1] F. F. Ferreira, H. P. S. Correa, M. T. D. Orlando, J. L. Passamai, Jr., C. G. P. Orlando, I. P. Cavalcante, F. Garcia, E. Tamura, L. G. Martinez, J. L. Rossi, F. C. L. de Melo, *J. Synchrotron Radiat.* **2009**, *16*, 48.

[2] A. Magnéli, *Acta Chem. Scand.* **1957**, *11*, 28.

[3] S. Shibata, Y. Hirose, A. Chikamatsu, E. Ikenaga, T. Hasegawa, *Appl. Phys. Lett.* **2020**, *117*, 111903.

[4] E. Reynolds, Z. Zhang, M. Avdeev, G. J. Thorogood, F. Poineau, K. R. Czerwinski, J. A. Kimpton, B. J. Kennedy, *Inorg. Chem.* **2017**, *56*, 9219.

[5] a) E. E. Rodriguez, F. Poineau, A. Llobet, A. P. Sattelberger, J. Bhattacharjee, U. V. Waghmare, T. Hartmann, A. K. Cheetham, *J. Am. Chem. Soc.* **2007**, *129*, 10244; b) A. L. Ivanovskii, T. I. Chupakhina, V. G. Zubkov, A. P. Tyutyunnik, V. N. Krasilnikov, G. V. Bazuev, S. V. Okatov, A. I. Lichtenstein, *Phys. Lett. A* **2005**, *348*, 66.

[6] I. Almahamid, J. C. Bryan, J. J. Bucher, A. K. Burrell, N. M. Edelstein, E. A. Hudson, N. Kaltsoyannis, W. W. Lukens, D. K. Shuh, H. Nitsche, T. Reich, *Inorg. Chem.* **1995**, *34*, 193.
